# Letter from the Editor in Chief

**DOI:** 10.19102/icrm.2023.14026

**Published:** 2023-02-15

**Authors:** Moussa Mansour


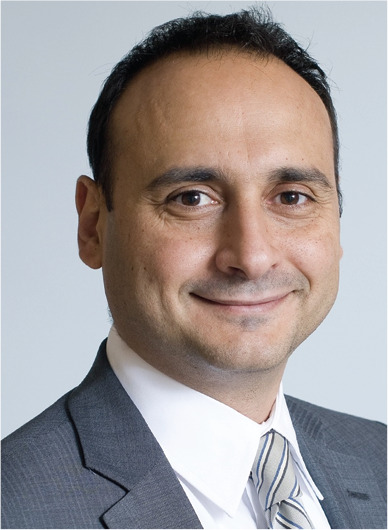


Dear readers,

The 28^th^ annual Atrial Fibrillation Symposium took place in Boston from February 2–4, 2023. During the conference, domestic and international faculty presented their most recent discoveries in all aspects of atrial fibrillation (AF) management. Although the meeting was very well attended and received, the late-breaking clinical trial (LBCT) session, during which 5 multicenter clinical trials were presented, garnered particular attention.

The first lecture of the session was presented by Dr. Vivek Reddy and titled “FLOW AF: A Multicenter Randomized Controlled Trial of Electrographic Flow-guided Ablation in Redo Patients with Non-paroxysmal Atrial Fibrillation.” The mapping system studied in this trial uses electrographic flow mapping, which enables full spatiotemporal reconstruction of electrical wavefront propagation to identify active AF sources and estimates the consistency of observed wavefront patterns. Under this approach, 1-min acquisitions were divided into 2-s segments, which were then collated together to identify active sources, and data were acquired using a commercially available 64-electrode basket catheter. Eighty-five patients with redo ablation for persistent and long-standing AF were enrolled in 4 centers in Europe. The acute primary endpoint was the ability to ablate the AF sources, and the secondary endpoint was AF recurrence. One instance of AF was induced, and patients were randomized to either re-isolation of the pulmonary veins alone or pulmonary vein isolation (PVI) + electrographic flow-guided ablation. Identification and ablation of AF sources were achieved in 95% of patients. During follow-up, freedom from AF was 51% improved in the EGF-guided ablation group compared to the PVI-alone group. The mean number of sources per patient was 1.3, and the mean ablation time was 10.2 ± 6.7 min. The majority of AF sources were localized to the left atrium.

I then presented data from the STELLAR study (“Pulmonary Vein Isolation of Paroxysmal Atrial Fibrillation with a Multi-electrode Radiofrequency Balloon Catheter: Results from the Global, Multicenter, STELLAR Study”) on behalf of my co-authors. This was a prospective multicenter single-arm study conducted at 36 sites in the United States, Europe, and China. The objective was to evaluate the safety and effectiveness of a radiofrequency multi-electrode 28-mm compliant balloon (HELIOSTAR™; Biosense Webster, Diamond Bar, CA, USA) integrated with the CARTO^®^ 3-dimensional electroanatomical mapping system (Biosense Webster) in patients with symptomatic drug-refractory paroxysmal AF. The balloon in question contains 10 electrodes used for mapping and ablation. The primary effectiveness endpoint was freedom from AF/atrial flutter (AFL)/atrial tachycardia (AT) lasting ≥30 s during the 12-month follow-up period. There was also a primary safety endpoint consisting of freedom from major AF ablation complications. Per protocol, a total of 238 patients were treated, and the primary endpoint was met, with 67.7% of patients remaining free from AF/AFL/AT at 12 months. The primary safety endpoint was also met, and the major complications included 1 death. Clinical success, which was defined as freedom from asymptomatic AF, and freedom from repeat ablations were achieved in 77% and 92% of patients, respectively. There was also an observed improvement in the quality of life and a reduction in anti-arrhythmic medication use and cardioversions.

The third LBCT lecture, titled “AF Ablation Using a Variable-loop Pulsed Field Ablation Catheter Integrated with 3D Mapping System: One-year Outcomes from inpIRE,” was also presented by Dr. Vivek Reddy. The ablation catheter used in this study was a circular catheter with 10 electrodes, enabling bipolar pulsed-field ablation at 1800 V. Enrolled patients had drug-refractory paroxysmal AF, and the primary effectiveness endpoint was freedom from documented AF/AT/AFL lasting ≥30 s at 12 months of follow-up. The study was stopped early because interim analysis showed that both the primary effectiveness and safety endpoints were met. In total, 186 subjects were enrolled, 83 of whom reached 12 months of follow-up. Entrance block was achieved in all patients. The procedure time was 70.1 ± 27.2 min, and there were no major safety events. In a subgroup of 33 patients, 4 (12%) had silent cerebral lesions. The primary effectiveness endpoint at 12 months was reached in 70.9%. Freedom from symptomatic recurrence was achieved in 78.9% of patients, and freedom from repeat ablation was achieved in 92.5%.

Next, Dr. Gery Tomassoni presented the “AcQForce Flutter Trial Clinical Results: Force-sensing RF Ablation with Low Flow Gold Tip Catheter for Typical Atrial Flutter” lecture. The objective of this study was to demonstrate the effectiveness of a 3.5-mm gold-tipped electrode catheter in the ablation of typical AFL. Gold is thought to have a superior thermal conductivity to that of other materials used in conventional ablation catheters. The primary safety endpoint was freedom from procedural complications, and the primary effectiveness endpoint was acute procedural success, which was defined as bidirectional block across the cavotricuspid annulus. The ablation system was integrated with the AcQMap mapping system (Acutus Medical, Carlsbad, CA, USA). In total, 115 patients were treated, and both the primary safety and effectiveness endpoints were met. There was 1 case of pericardial effusion, 2 cases of pericarditis, and 12 groin hematomas. Successful bidirectional block was achieved in 94% of patients.

The last LBCT lecture, presented by Dr. Vivek Reddy, was titled “AF Ablation Using a Novel Single-shot Map-and-ablate Spherical Array Pulsed Field Ablation Catheter: Impact of Application Repetition on Durability of Pulmonary Vein Isolation.” The ablation system in this trial consisted of a spherical array 30 mm in diameter with 122 mapping and ablation electrodes integrated with a 3-dimensional electroanatomical mapping system. Fifty-nine patients were enrolled in this prospective first-in-human study conducted in the Czech Republic. The procedure time was 82 ± 29 min. During remapping studies performed at 3 months after the index procedure, durable PVI was achieved in 99% of pulmonary veins. There was 1 pericarditis event in 1 patient, and a 5% rate of silent cerebral ischemia was recorded.

The above-mentioned studies investigated different technologies for AF ablation, and all met their primary safety and effectiveness endpoints with excellent results. Also, most of the ablation systems described in these LBCTs appear to be user-friendly and likely to facilitate the ablation procedure. It is expected that they will have a significant impact on the ablation landscape, and we are looking forward to having them available for use in the United States. The detailed presentations and other content from the AF Symposium will be available soon on the conference website (https://www.afsymposium.com).


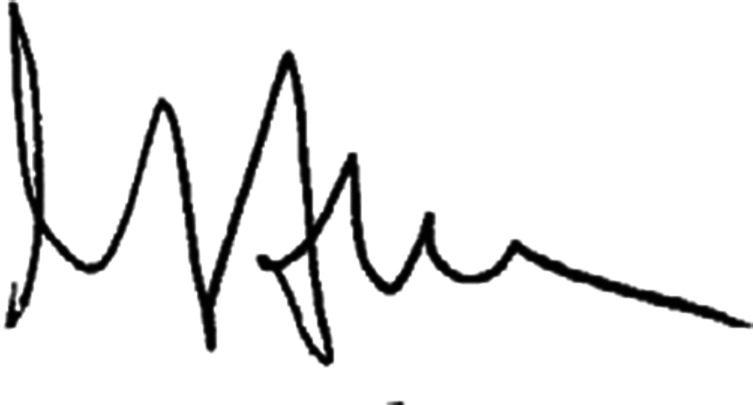


Sincerely,

Moussa Mansour, md, fhrs, facc

Editor in Chief

*The Journal of Innovations in Cardiac Rhythm Management*

MMansour@InnovationsInCRM.com

Director, Atrial Fibrillation Program

Jeremy Ruskin and Dan Starks Endowed Chair in Cardiology

Massachusetts General Hospital

Boston, MA 02114

